# Cervical cancer information access and dissemination strategies among rural Kenyan women: A mixed methods study

**DOI:** 10.4102/jphia.v17i1.1457

**Published:** 2026-01-27

**Authors:** Joyline Chepkorir, Lucy Kivuti-Bitok, Nancy Perrin, Deborah Gross, Joseph J. Gallo, Jean Anderson, Nancy R. Reynolds, Susan Wyche, Hillary Kibet, Vincent Kipkuri, Anastasha Cherotich, Dominique Guillaume, Hae-Ra Han

**Affiliations:** 1 Department of Community Public Health, College of Nursing, Johns Hopkins University, Baltimore, United States of America; 2 Department of Nursing Sciences, Faculty of Nursing, University of Nairobi, Nairobi, Kenya; 3 Department of Biostatistics, College of Nursing, Johns Hopkins University, Baltimore, United States of America; 4 Center for Equity in Child and Youth Health and Wellbeing, College of Nursing, Johns Hopkins University, Baltimore, United States of America; 5 Department of Mental Health, School of Public Health, Johns Hopkins University, Baltimore, United States of America; 6 Department of Gynecology and Obstetrics, School of Medicine, Johns Hopkins University, Baltimore, United States of America; 7 Center for Global Initiatives, School of Nursing, Johns Hopkins University, Baltimore, United States of America; 8 Department of Media and Information, College of Communication, Arts and Sciences, Michigan State University, East Lansing, United States of America; 9 Department of Global Health, Faculty of Global Community Health and Travel Medicine, Masinde Muliro University of Science and Technology, Kakamega, Kenya; 10 Department of Public Health, School of Epidemiology and Biostatistics, Masinde Muliro University of Science and Technology, Kakamega, Kenya; 11 Department of Infectious Disease, School of Nursing, Johns Hopkins University, Baltimore, United States of America; 12 Department of Health, Behavior, and Society, School of Nursing, Bloomberg School of Public Health, Johns Hopkins University, Baltimore, United States of America

**Keywords:** cervical cancer, screening, information dissemination, rural, women

## Abstract

**Background:**

Cervical cancer (CC) remains a leading cause of cancer-related deaths among women in sub-Saharan Africa. In rural Kenya, screening uptake is particularly low, partly because of limited access to reliable health information and other structural barriers such as the unavailability of screening equipment and the cost of screening.

**Aim:**

To examine the relationship between access to CC information and screening uptake, and to identify information needs, preferences and dissemination strategies among women with low educational attainment in resource-limited rural settings.

**Setting:**

Rural communities in Bomet and Kericho Counties, Kenya.

**Methods:**

We conducted a mixed methods study among 174 women recruited through purposive convenience sampling. Data collection involved interviewer-administered cross-sectional surveys and semi-structured interviews (*n* = 21). Quantitative data were analysed using logistic regression, and qualitative data using thematic analysis. Findings were integrated during interpretation.

**Results:**

Participants had a mean age of 45 years; 88.5% were from low-income households. Although 82.2% had heard of CC – primarily via news media (36.8%), health workers (24.1%) and social networks (21.3%) – only 6.3% had been screened. Health workers and the radio were the most trusted information sources. Access to each additional information source was associated with 4.66 times higher odds of screening (95% confidence interval: 1.19–18.25). Despite this, 92% felt inadequately informed. Integrated findings underscored the need for culturally relevant, literacy-sensitive approaches.

**Conclusion:**

Screening uptake remains low but improves with exposure to diverse, trusted information sources.

**Contribution:**

This study highlights the importance of tailored, community-based strategies to enhance CC screening in underserved rural populations.

## Introduction

Globally, cervical cancer (CC) is the fourth most commonly diagnosed cancer among women,^1^ with sub-Saharan Africa (SSA) bearing the highest regional incidence and mortality rates.^2^ In Kenya, CC is the leading cause of cancer-related deaths, responsible for approximately 3200 deaths in 2020.^2^ Cervical cancer is primarily caused by persistent infection with high-risk human papillomavirus (HPV) types, mainly types 16 and 18, which together account for about 70% of cases worldwide.^3^ Although HPV vaccination and routine screening are highly effective in preventing CC, uptake remains low in SSA largely because of the limited availability of vaccination and screening programmes.^3,4^ While CC screening has helped address gaps caused by missed opportunities for HPV vaccination, particularly during adolescence when the vaccine is most effective, a large proportion of eligible women in SSA have never been screened.^5^ The 2022 Demographic Health Survey administered in 11 countries showed that only 10% of women had undergone CC screening, which is well below the World Health Organization’s (WHO) 2030 target of 70% screening coverage.^6,7^

Women in rural SSA face the greatest barriers to screening, including limited access to health facilities offering CC screening, high cost of screening, belief in traditional medicine and low risk perception.^8,9^ Screening rates in rural SSA range from 0.4% to 14% compared to 2% to 20% in urban areas.^10^ In Kenya, rural screening uptake is estimated at just 2.6%, significantly lower than the 4% observed among urban women.^11^ Limited access to accurate and reliable CC information is a key barrier to prevention.^12,13^ Inadequate information contributes to stigma, misconceptions and delays in screening and treatment.^14^ Among women with low educational attainment in rural areas, the sources and modes of health communication play a critical role in shaping CC literacy and screening uptake, which is often lower compared to their more educated counterparts.^15,16^ While targeted mass media campaigns have been proposed as more effective strategies for rural women in SSA, there are mixed results on their impact on CC screening uptake.^16^ Additionally, little research has examined the informational needs, preferences and impact of communication strategies on screening uptake among rural women with low educational attainment. To achieve the WHO’s 70% screening goal, SSA countries must scale up screening through culturally tailored interventions that account for structural and informational barriers.^7^

Addressing these gaps requires both quantitative and qualitative inquiry. A mixed methods approach offers a comprehensive assessment, quantifying the relationship between information access and screening uptake while exploring women’s lived experiences, informational needs and culturally appropriate dissemination strategies. Such insights are essential for designing effective interventions to increase CC screening uptake in rural Kenya and other SSA contexts. The aims of this study were to (1) examine the relationship between rural Kenyan women’s access to CC information and screening uptake, and (2) explore CC information needs, preferences and strategies for effective information dissemination in resource-limited settings.

### Theoretical framework

**FIGURE 1:**
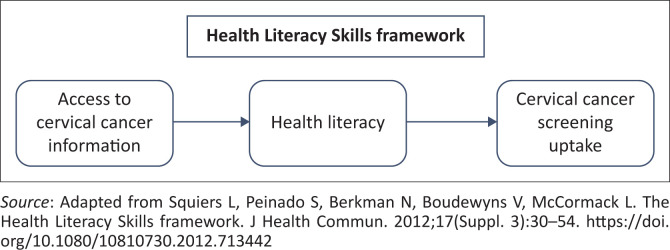
Conceptual framework.

This study was guided by the Health Literacy Skills (HLS) framework (Figure 1),^17^ which examines how individuals access, process and apply health information to make informed decisions. The framework highlights four main components: (1) factors influencing the development and use of HLS (e.g. demographics, prior knowledge, resources); (2) health-related stimuli (e.g. sources and channels of health information); (3) the HLS required to comprehend and apply information; and (4) mediators linking health literacy to health outcomes.

In this study, the first three components were adapted to examine how rural Kenyan women access CC information and how these factors relate to screening uptake. Health literacy was conceptualised as a potential mediator between information exposure and screening behaviour. The HLS framework also informed our use of a mixed methods design to achieve a comprehensive understanding of both the measurable associations and the subjective experiences shaping women’s health behaviours.

## Research methods and design

### Study design

This was a cross-sectional study using a convergent mixed methods design to comprehensively assess CC information access and screening uptake among rural Kenyan women. Quantitative data were collected from 174 participants through interviewer-administered surveys. Qualitative data were obtained from a purposive sub-sample (*n* = 21) of survey participants through semi-structured interviews. Quantitative and qualitative findings were integrated during interpretation to enhance contextual understanding.

### Study setting

The study was conducted in Bomet and Kericho Counties. Both counties are predominantly rural, with 96.8% of Bomet’s population and 89.6% of Kericho’s population residing in rural areas.^18^ These areas are characterised by smallholder farming, with most residents engaged in subsistence agriculture, tea farming and livestock rearing as primary sources of livelihood. Health facilities in both counties consist mainly of dispensaries and health clinics, with referral hospitals.^19^ However, access to specialised health services, including cancer screening and treatment, remains limited, requiring residents to travel to larger urban centres such as Nairobi.^20^ The population is predominantly from the Kipsigis subgroup of the Kalenjin ethnic community, with culturally embedded practices and beliefs influencing health-seeking behaviours. Kipsigis is the primary language spoken. Similar to other women in rural Kenya, women in these counties face challenges such as illiteracy or low literacy rates, limited access to health information, and inadequate healthcare infrastructure.^21,22^ The setting’s rural nature, coupled with socio-economic and health system constraints, provides a critical context for understanding access to CC information, health literacy and screening behaviours among women in these counties.

### Inclusion and exclusion criteria

Eligible participants were women who: had completed Grade 8 or lower, self-identified as female, were eligible for CC screening (aged 18–65 years with no history of total hysterectomy), and were proficient in the Kipsigis language. Participants were excluded if they had an acute or terminal illness or cognitive impairments that would limit their ability to participate meaningfully.

### Participant recruitment and sampling

Trained local research staff recruited participants from homes, churches and health centres, with support from village leaders and trusted community members, including church leaders. Convenience sampling was used for survey enrolment. For qualitative interviews to capture diverse perspectives, purposive maximum variation sampling was employed based on participants’ age, CC awareness, screening status and access to CC information.

### Data collection and management

Survey instruments and interview guides were developed in English using validated tools and prior studies and interview guide.^23,24,25,26^ Instruments were translated into Kipsigis and back-translated into English to ensure accuracy. Bilingual registered nurses and a local gynaecologist assessed the tools for face validity and cultural appropriateness. Pilot testing with 24 participants led to minor revisions for clarity and cultural sensitivity. Quantitative data were collected using the REDCap Mobile Application. Midway through data collection, a protocol amendment was made to address knowledge gaps identified among participants. An information sheet summarising key facts about CC was introduced, and research assistants reviewed this information with each participant after administering the survey, especially for those with limited literacy.

Semi-structured interviews were conducted in Kipsigis, audio-recorded, and focused on barriers and facilitators to CC screening, trusted information sources, and preferred communication methods. Interviews lasted approximately 45 min to 60 min. Interviewers fostered open discussions to elicit rich, reflective responses. Participants received an incentive of 330.00 Kenyan shillings (KSh) for completing the survey, and an additional KSh 330.00 (approximately $2.31) for participating in interviews, in appreciation of their time and contributions.

### Measures

Participants who had heard about CC were asked to identify their information sources, including health workers (nurses, community health workers, or doctors), news media (radio or television), social networks (family, friends or neighbours), religious leaders, teachers, printed materials, or others. Multiple responses were allowed. Screening status was assessed with a binary (yes/no) question: ‘*Have you ever been screened for cervical cancer in your lifetime?*’

Health literacy was measured using the 12-item Health Literacy Test for Limited Literacy (HELT-LL) populations.^27^ Sample questions include: ‘if you are worried about a health problem, do you usually ask your friends and neighbours first for information and advice before going to the clinic?’ and ‘how often do you need to have someone help you when you read instructions, pamphlets, or other written material from your doctor/nurse/pharmacist?’

Cronbach’s alpha was 0.60 in the original study. In our sample, four items with Cronbach’s alpha < 0.49 were removed (Online Appendix 1), resulting in an 8-item scale with a Cronbach’s alpha of 0.57 within the acceptable range.^28^ The remaining items assessed numeracy, print, critical and communicative literacy. Total scores ranged from 0 to 8. Participants scoring below the mean (4.2) were categorised as having inadequate health literacy.

### Sample size calculation

The sample size estimation assumed that 50% of women had no exposure to CC information.^29^ Based on an anticipated screening rate of 1.6% among the unexposed and 14% among the exposed,^11,30^ a sample of 140 participants was required to achieve 80% power at α = 0.05.^31,32^ To account for potential non-response, we aimed to recruit at least 150 participants.

### Statistical analysis

The final sample included 150 survey respondents and a sub-sample of 24 participants from the survey pilot (total *N* = 174). Descriptive statistics (means, standard deviation [s.d.], medians, ranges, frequencies and percentages) were used to summarise participant characteristics. Bivariate logistic regression was used to examine associations with the outcome. No covariates were significantly associated with CC screening in bivariate analysis; thus, multivariable regression was not performed. Analyses were conducted using STATA/BE (version 17).

For qualitative data, interviews were transcribed verbatim in Kipsigis and coded by four bilingual coders using Dedoose. A combination of inductive and deductive thematic analysis was employed.^33^ Coding was conducted in Kipsigis to preserve cultural meaning, with each transcript independently coded by two coders. Themes were discussed iteratively. Translated English summaries were produced for reporting. Quantitative and qualitative results were integrated using joint display techniques.^34^

### Ethical considerations

Ethical clearance to conduct this study was obtained from the Johns Hopkins University, National Commission for Science, Technology & Innovation (NACOSTI/P/23/23289, Ref: 774947), the Johns Hopkins Medicine Human Subjects Research Institutional Review Board (IRB00357410) and the Amref Ethical and Scientific Review Committee in Kenya (REF: AMREF-ESRC P1394/2023). Verbal informed consent was obtained from all participants prior to data collection. Interviews were conducted in Kipsigis, with participants given the opportunity to ask questions before consenting. To maintain confidentiality, no identifying information was collected. All data collection devices were password-protected, and audio files were securely uploaded to a cloud-based database and deleted from recording devices after transcription.

## Results

Table 1 presents the characteristics of the survey sample and the qualitative sub-sample. Most participants were middle-aged (mean age = 45.3 years, s.d. = 13.2), married (83.3%), and residents of Bomet County (64.4%). Over half of the participants (51.7%) had completed formal education between grades 4 and 8, and most were self-employed (77%). A large proportion (88.5%) reported a monthly household income of ≤ $35.00, with 91.4% indicating that this amount did not adequately meet household needs. Most participants rated their health as good (63.6%), were uninsured (75.3%) and made their own healthcare decisions (79.9%). Travel time to the nearest health facility ranged from 30 min to 2 h for the majority (54%). Regarding access to information, 69.5% owned a non-smartphone, and 43.7% owned a radio. Characteristics of the qualitative sub-sample are also detailed in Appendix 1 – Table 1-A1.

**TABLE 1:** Sample characteristics (*N* = 174).

Characteristic	*n*	%	Mean	s.d.
**Age (years)**	-	-	45.3	13.18
**Marital status**				
Married	145	83.30	-	-
Unmarried	29	16.70	-	-
**County of residence**				
Bomet	112	64.40	-	-
Kericho	62	35.60	-	-
**Education**				
No formal education	32	18.40	-	-
Grades 1–3	52	29.90	-	-
Grades 4–8	90	51.70	-	-
**Employment status**				
Self-employed	134	77.00	-	-
Unemployed	34	19.50	-	-
Employed in the public or private sector	5	2.90	-	-
Missing	1	0.57	-	-
**Household monthly income**				
≤ $35.00	154	88.50	-	-
$36.00–$142.00	20	11.50	-	-
**Income comfortably meets household needs**				
Yes	15	8.60	-	-
No	159	91.40	-	-
**Insurance status**				
Insured	41	23.60	-	-
Uninsured	131	75.30	-	-
Missing	2	1.10	-	-
**Health status**				
Poor or fair	65	36.40	-	-
Good or very good	109	63.60	-	-
**Access to health information sources†**				
Owns radio	76	43.70	-	-
Owns television	19	10.90	-	-
Owns a mobile phone	121	69.50	-	-
Owns smartphone	15	8.60	-	-
None	16	9.20	-	-
**Travel time to the nearest health facility**				
< 30 min (reference)	76	43.70	-	-
30 min–120 min	94	54.00	-	-
Missing	4	2.30	-	-
**Healthcare decisions†**				
Self	139	79.90	-	-
Self and spouse	12	6.90	-	-
Mother or spouse	7	4.00	-	-
Missing	16	9.20	-	-

s.d., standard deviation.

† , Select all that apply item.

### Cervical cancer information access, needs and preferences for dissemination

Table 2 summarises the participants’ access to CC information, needs and preferences for CC information dissemination. The majority of participants had heard of CC (82.2%), mainly from the news media (36.8%), followed by health workers (24.1%) and social networks such as family or friends (21.3%). Few participants cited teachers or religious leaders (1.7%) as sources. Among those who had heard of CC (*n* = 143), only 39.9% demonstrated good knowledge. Most women (93.7%) reported never having been screened for CC, and 92% felt uninformed about the disease. Reasons for not screening are summarised in Appendix 1 – Table 2-A1.

**TABLE 2:** Cervical cancer information access, needs and preferences for dissemination.

Characteristic	*n*	%
**Heard of CC**		
Yes	143	82.2
No	29	16.7
Missing	-	0.6
**Sources of CC information†**		
News media (television and radio)	64	36.8
Social networks	37	21.3
Healthcare workers	42	24.1
Other (teachers, religious leaders)	3	1.7
**CC screening status**		
Yes	11	6.3
No	163	93.7
**Well-informed about CC**		
Yes	14	8.0
No	160	92.0
**Actively searched for CC information**		
Yes	84	48.3
No	90	51.7
**Sources searched for CC information†**		
Radio	65	37.4
Television	3	1.7
WhatsApp	1	0.6
Facebook	1	0.6
Friends	3	1.7
Family	4	2.3
Neighbour(s)	1	0.6
Health worker(s)	65	37.4
Church community	5	2.9
Women group	5	2.9
**Interested in learning more about CC**		
Yes	147	84.5
No	25	14.4
Missing	2	1.1
**Preferred CC information sources†**		
CHW	30	17.2
Nurse/Doctor	163	93.7
Radio	88	50.6
Television	7	4.0
Women’s leader or village leader	18	10.3
WhatsApp or Facebook	3	1.7
Social networks	56	32.2
Other	3	1.7
**Trusted sources of CC information†**		
Radio	51	29.3
Television	8	4.6
Church leader	8	4.6
Government administrator(s)	2	1.2
SMS	1	0.6
CHW	20	11.5
Nurse or doctor	165	94.8
Herbalist(s) or other	3	1.7
**Preferred CC information delivery channels†**		
Verbal	163	93.7
Videos	22	12.6
Pictures	20	11.5
**Preferred settings**		
Health facility	70	40.2
Anywhere	50	28.7
Community setting	36	20.7
Home	16	9.2
Unknown	2	1.2

Note: Sub-sample (*n* = 143).

SMS, short message services; CHW, community health worker; CC, cervical cancer.

† , Select all that apply item.

Less than half of the participants (48.3%) had actively searched for CC-related information. When asked about their preferred and trusted sources for receiving CC information, the most frequently endorsed sources were nurses and doctors, followed by the radio. The television, mobile applications (WhatsApp or Facebook) and short message services (SMS) were least preferred. Verbal communication was considered the most effective method for delivering CC information (93.7%), followed by videos (12.6%), and pictures (11.5%) were the least preferred. Preferred locations for receiving CC information were health facilities (40.2%), followed by community settings (20.7%).

### Associations among health literacy, cervical cancer information sources and cervical cancer screening

Bivariate analysis indicated that among participants who had heard about CC (*n* = 143), the odds of ever being screened were 4.66 times higher for each additional source of CC information accessed (OR = 4.66, 95% confidence interval [CI]: 1.19–18.25). Health literacy was not significantly associated with lifetime CC screening among this subgroup (OR = 1.39, 95% CI: 0.95–2.02). Therefore, mediation analysis to test health literacy as a mediator between information sources and screening uptake was not conducted.

### Qualitative results

The qualitative findings underscore the pivotal role of accessible and trusted CC information in promoting screening uptake among rural women. Participants frequently cited health workers, radio broadcasts and community leaders as the primary sources that motivated their decision to screen – often marking their first meaningful exposure to the severity of CC and the importance of early detection. Many also voiced a strong need for comprehensive CC education, covering causes, symptoms, prognosis, treatment options and where to access services. Preferences for how, by whom, and where this information should be communicated were clear, highlighting a deep appreciation for verbal, face-to-face messaging delivered by trusted figures in accessible and private settings. These themes are elaborated below.

### Access to cervical cancer information enhances screening uptake

Access to timely and relatable CC information significantly influenced screening behaviour. Most women who underwent screening attributed their decision to persuasive messages from health professionals, media or local leaders. A radio report on the death of a prominent leader because of CC, for example, sparked fear and awareness in one participant, prompting her to seek screening:

‘… I had heard on the radio that the [*government leader’s position*] had succumbed to CC. You know that made me afraid […] I learned that it is important for one to get screened so that it (CC) can be detected early and treated. Before then, I didn’t know about cancer … that it kills people’ (ID07, age 33, female).

### Rural women’s cervical cancer information needs

Across the sample, women expressed a desire for detailed, CC-literacy-focused education. They sought to understand what causes CC, how it manifests, whether it is treatable and where services are available. This educational gap was articulated by a participant in her early 30s:

‘I am interested in knowing what causes this disease, how a woman can know if she has CC and if a person can be treated once they are screened and they are diagnosed with it.’ (ID07, age 31, female)

### Preferences for cervical cancer information dissemination

Participants articulated specific preferences regarding the source, format and setting of CC information:

**Trusted Messengers**: Women preferred information from familiar and authoritative figures, such as health workers, community leaders and religious leaders. Face-to-face delivery enhanced credibility and allowed for immediate clarification. In contrast, SMS messages were viewed with scepticism, often ignored or not taken seriously:

‘… some would just look at the SMS notification and ignore it … But when you go to someone directly, they will understand that the disease [*CC*] is serious.’ (ID07, age 33, female)

**Preferred Formats:** Verbal communication was favoured, especially among those with limited literacy or without smartphones. Videos were seen as useful complements, while illustrations helped with anatomical understanding:

‘Verbal information is good for educating illiterate people.’ (ID139, age 53, female)

**Accessible Settings**: Preferred venues ranged from hospitals and community gatherings to individual homes. However, the presence of a trusted health worker often mattered more than the setting itself:

‘… So, when there is a qualified healthcare professional, […] it is good to meet people as a group. Otherwise, I feel that it [*CC education*] should reach me at the hospital.’ (ID124, age 40, female)

### Strategies for effective cervical cancer information dissemination

Four actionable strategies emerged to guide effective and inclusive CC information dissemination in rural contexts:

**Leverage Trusted Messengers and Channels**: Health workers, CHWs and local leaders (e.g. chiefs, clergy) were viewed as the most credible messengers. Trusted channels included radio and in-person communication, while SMS and official alerts were often dismissed:

‘I trust information from … someone from this village, for instance, a chief …’ (ID07, age 33, female)

**Health-Worker-Led Education Coupled with Screening**: Participants favoured integrated approaches where health workers provided both education and immediate access to screening – enhancing trust and convenience:

‘… a health care worker [*providing cancer education*] at the hospital would be preferable since one would also have access to screening by the doctor or a nurse ….’ (ID101, 52 years, female)

**Multi-Channel and Community-Based Outreach**: To ensure wide coverage, participants suggested blending traditional (e.g. radio) and digital platforms (e.g. WhatsApp, Facebook) with community outreach, especially for women relying on traditional medicine or facing mobility barriers:

‘… send health workers to educate people in the community … many women use herbal medicines, so hospital-based information wouldn’t reach them.’ (ID40, age 45, female)

**Tailored Delivery to Balance Reach and Privacy**: While group education in churches or schools allowed for broad reach, many women valued private, home-based visits to discuss sensitivities:

‘… if they come to my home, then it is better as there is privacy … in a public place like a school … you cannot ask a question that you want to ask.’ (ID16, age 38, female)

This highlights the need for flexible strategies that respect both social dynamics and individual comfort levels.

### Integration

The integrated findings from this mixed methods study highlight the critical role of CC information in promoting screening uptake among rural women. Quantitative data showed that each additional increase in access to CC information was associated with over fourfold higher odds of being screened. Qualitative findings reinforced this association. Women commonly described being motivated to seek screening after receiving information from trusted health workers, radio broadcasts or community leaders. Despite this exposure, however, more than 90% still felt inadequately informed about CC – especially regarding its causes, symptoms, treatment options and prognosis.

These knowledge gaps point to the need for ongoing, CC-literacy-focused education. Participants favoured clear, verbal communication delivered by trusted messengers, particularly health workers. They also preferred receiving information in familiar and accessible settings such as health facilities, homes and community gatherings. Additionally, the integration of findings emphasised the importance of using multiple communication channels. A combination of traditional media (like radio and community meetings) and digital platforms (such as WhatsApp and Facebook) can help extend the reach of CC information while accommodating different preferences and access levels. Together, these findings support the implementation of community-engaged, health-worker-led interventions. Such approaches should prioritise trust-building, bridge persistent knowledge gaps, and adapt delivery methods to local contexts to ensure both effectiveness and sustainability. Table 3 shows a summary of integrated qualitative and quantitative results.

**TABLE 3:** Integration of quantitative and qualitative results.

Quantitative findings	Qualitative and illustrative quotes	
	Themes	Quotes
Every additional increase in access to CC information was significantly associated with 366% greater odds of CC screening (OR = 4.66; CI = 1.19-18.25).	Access to CC information enhances CC screening uptake	‘When the doctor announced screening and health workers came to the dispensary, I decided to go so I could know my health status.’ (ID101, age 52, female)
92% of participants felt inadequately informed about CC.	Rural Women’s CC Information Needs	‘I no longer remember the information I heard before. That’s why I say those who know should come and educate us.’ (ID74, age 31, female) ‘I would want to know, for example, if I’m diagnosed, can I fully recover and live a normal life?’ (ID16, age 38, female) ‘Once the screening is done and the disease is found, are there medications?’ (ID124, age 40, female)
**Preferences for CC information dissemination**		
Most women preferred doctors or nurses (93.7%), the radio (50.6%), their social networks (32.2% each), CHWs (17.2%), women’s leader/village leader (10.3%), WhatsApp/Facebook (1.7%), or other sources (1.7%).	Trusted messengers	‘I trust a doctor or a nurse to deliver CC information because they are trained.’ (ID16, age 38, female)
93.7% of women would prefer verbally delivered CC information, followed by videos (12.6%), then pictures (11.5%).	Preferred formats	‘I want verbal communication. With this disease, I need to ask the healthcare provider more during my next checkup.’ (ID16, age 38, female) ‘Verbal information is good for anyone without a phone.’ (ID124, age 40, female) ‘Videos would be better because people can listen and watch.’ (ID74, age 31, female) ‘If someone just sends an SMS about CC, I won’t believe it – I’ll question their intentions.’ (ID49, age 54, female) ‘I prefer pictures because I can’t read or write.’ (ID54, age 48, female)
The majority (40.2%) of participants would prefer health facilities, followed by community settings (20.7%), and participants’ homes (9.2%).	Accessible settings	‘CC information should be shared everywhere – radio, Facebook, WhatsApp – so everyone can access it.’ (ID23, age 45, female) ‘Look for a well-known place where people can gather.’ (ID07, age 33, female) ‘I prefer where I live. That way, others who don’t want to go to the hospital can also get the information.’ (ID23, age 45, female) ‘A nearby hospital is better. If it’s in a school, you still need to find a health worker for screening.’ (ID74, age 31, female)
**Strategies for effective CC information dissemination**		
Most women trusted healthcare workers (94.8%), the radio (29.3%), church leaders and TV (4.6% each), herbalists or other sources (1.7%), government administrators (1.2%), and lastly short message services (SMS) (0.6%).	Leverage Trusted Messengers and Channels	‘It’s good to gather women by neighbourhoods and invite health workers. Unlike rushed radio programmes, health workers make sure you understand the information.’ (ID101, age 52, female)
84.5% were interested in learning more about CC and 93.7% had never been screened.	Health-Worker-Led Education Coupled with Screening	‘Health workers should reach every rural area, gather people, and educate them so they understand and agree to be screened.’ (ID101, age 52, female)
28.7% of women would prefer being educated on CC anywhere, 20.7% in community settings and 9.2% in home settings.	Multi-Channel and Community-Based Outreach	‘I especially value health education from hospital providers. We women also enjoy TV programmes – especially those that give hope after a CC diagnosis.’ (ID146, age 32, female) ‘Community gatherings are effective. Start with one village and inform them of the CC education session. Move to the next village after that. This way, many people are reached.’ (ID108, age 50, female)
92% felt inadequately informed about cervical cancer, 51.7% were not actively searching for CC information and 84.5% were interested in learning more about CC.	Tailored Delivery to Balance Reach and Privacy	‘CC education can be included in women’s gatherings. These meetings are a good opportunity.’ (ID117, age 50, female)

OR, odds ration; CI, confidence interval; CHW, community health worker; TV, television; CC, cervical cancer.

## Discussion

This mixed methods study explored access to CC information, informational needs, preferences and dissemination strategies among rural Kenyan women with low educational attainment. Findings partially supported our hypotheses: increased access to diverse CC information sources was significantly associated with higher screening uptake, although health literacy did not mediate this relationship. Integration of quantitative and qualitative findings revealed strong convergence, enhancing the contextual understanding of screening behaviours. Despite relatively high CC awareness of about 82.2%, screening uptake remained very low at 6.3%. This awareness-screening gap mirrors findings from previous studies in rural Kenya and other parts of SSA.^11,35,36^ Subjective perceptions of knowledge were also low; 92% of participants reported feeling inadequately informed. Qualitative results highlighted critical gaps in CC-specific knowledge, including understanding of causes, symptoms, risk factors, available screening services and treatment options. Navigational health literacy, such as knowing where and how to access screening, emerged as a key barrier to screening uptake.

Quantitative analysis showed that each additional CC information source accessed was associated with a fourfold increase in the odds of screening. This finding aligns with evidence from rural Ethiopia, where women who accessed CC information were over 10 times more likely to screen.^37^ These results emphasise that exposure to multiple trusted information sources can compensate for limited formal education and health literacy, promoting better screening behaviours. Besides, interventions to improve CC literacy and screening uptake must be culturally tailored, literacy-sensitive and community-based.

Women in this study overwhelmingly preferred verbal communication from trusted health professionals. This preference is consistent with broader research indicating that interpersonal communication with healthcare workers and community health workers is highly valued across populations, regardless of literacy level.^15,38^ However, workforce limitations pose challenges for scaling such approaches. Kenya’s recent deployment of over 100 000 Community Health workers offers a promising avenue for expanding CC education and outreach efforts.^39^

Local radio also emerged as a highly trusted and accessible channel for CC information dissemination. This finding supports previous literature from other resource-limited and rural settings in SSA.^16,40^ A multi-country survey of 72 565 rural women in SSA revealed that a higher proportion of those who have tested for CC do not watch television (49.36%) nor read newspapers or magazines (75.96%). However, most of them reported listening to the radio at least once a week (52.11%), underscoring the platform’s broad reach and relevance for health communication in rural communities.^16^ Given widespread radio ownership even among low-income rural populations, radio programmes could be leveraged for CC education and mobilisation campaigns, particularly among women with limited access to digital technologies. In addition to trusted messengers and channels, participants emphasised the importance of delivery formats and settings that respect privacy and enhance engagement. Verbal, face-to-face communication was preferred, supplemented by visual aids such as videos and pictures when appropriate. Participants also valued receiving CC information both at health facilities and in community settings, with some preferring home-based outreach to preserve privacy and allow for sensitive discussions. These findings highlight the need for flexible, multi-channel dissemination strategies that accommodate diverse preferences and access barriers.

### Limitations

This study has some limitations. Firstly, its cross-sectional design precludes causal inference. Secondly, reliance on self-reported data introduces the potential for recall bias. Thirdly, the Health Literacy Test for Limited Literacy (HELT-LL) demonstrated moderate internal consistency reliability (α = 0.57) even after item reduction; however, no better-validated alternative existed for this context, and the tool was retained for comparability with prior studies. Fourthly, the actual screening uptake (6.3%) was lower than expected based on prior estimates (14%), which may have reduced the power to detect associations involving health literacy. Fifthly, qualitative interviews did not capture participants’ preferences for specific radio programmes, broadcasters or social media channels, limiting insights into message delivery optimisation. Lastly, the use of a convenience sampling strategy and recruitment through community gatekeepers (i.e. church and village leaders) may have introduced selection bias, limiting the representativeness of the sample and the generalisability of findings to broader rural populations, especially those less engaged with formal or community structures.

Despite these limitations, this study has notable strengths. It is among a few studies in SSA to quantitatively assess health literacy in a low-education rural sample and examine its relationship with CC information exposure and screening behaviours. The mixed methods design enriched understanding by integrating subjective experiences with objective associations, enhancing the credibility and applicability of the findings. Methodological rigour, including culturally appropriate tool adaptation, bilingual data collection, and robust thematic analysis, further strengthens the validity of the results.

## Conclusion

Our findings highlight the critical role of multi-source, culturally tailored CC information dissemination in improving screening uptake among rural Kenyan women. Oral communication delivered by trusted health workers remains essential, but leveraging local radio and expanding community-based outreach can significantly enhance reach and impact. Educational efforts should prioritise topics identified as key knowledge gaps, including CC causes, symptoms, risk factors, screening options and treatment pathways.

Scaling culturally adapted CC education initiatives through Kenya’s newly formalised community health workforce could accelerate progress towards national and global CC elimination targets. Future research should evaluate the effectiveness of multi-channel, trust-based CC communication interventions and explore the role of emerging technologies in reaching underserved rural populations.

## Appendix 1

**TABLE 1-A1:** Qualitative sample (*n* = 21) characteristics.

Characteristic	*n*	%	Mean	s.d.
**Age (years)**	-	-	45.4	10.8
**Marital status**			-	-
Married	16	76.00	-	-
Unmarried	5	23.80	-	-
**County of residence**			-	-
Bomet	14	66.70	-	-
Kericho	7	33.30	-	-
**Education level**			-	-
No formal education	4	19.10	-	-
Grade 1–3	5	23.80	-	-
Grade 4–8	12	57.10	-	-
**Employment status**			-	-
Self employed	18	85.70	-	-
Unemployed	3	14.30	-	-
**Household income/month**			-	-
≤ $35.00	18	85.70	-	-
$36.00–$70.00	2	9.50	-	-
$252.00–$322.00	1	4.80	-	-
**Income meets household needs**			-	-
Yes	1	4.80	-	-
No	20	95.20	-	-
**Insurance status**			-	-
Insured	4	19.00	-	-
Uninsured	17	81.00	-	-
**Health status**			-	-
Poor	10	47.60	-	-
Fair	11	52.40	-	-
**CC screening (*n* = 13)**			-	-
Screened	3	23.10	-	-
Never screened	10	76.90	-	-
**Actively searched for CC information**			-	-
Yes	11	52.40	-	-
No	10	47.60	-	-
**Sources of CC information searched**			-	-
Radio	1	4.80	-	-
Television	1	4.80	-	-
church community	1	4.80	-	-
CHW	3	14.30	-	-
Doctor	7	3.30	-	-
**Willingness to self-sample for HPV testing**			-	-
Yes	18	85.70	-	-
No	3	14.30	-	-
**Information about CC**			-	-
Well-informed	4	19.00	-	-
Feels uninformed	17	81.00	-	-
**Interested in learning more about CC**			-	-
Yes	19	90.50	-	-
No	2	9.50	-	-
**Preferred sources of CC information**			-	-
Radio	8	38.10	-	-
Women’s leader	3	14.30	-	-
WhatsApp	1	4.80	-	-
Facebook	1	4.80	-	-
Friends	1	4.80	-	-
Relatives	1	4.80	-	-
Health workers (nurse, doctor)	21	100.00	-	-
CHW	4	19.10	-	-
Church	3	14.30	-	-
Village leader	2	9.50	-	-
**Trusted sources of CC information**			-	-
Doctor	20	95.25	-	-
Nurse or CHW	7	0.33	-	-
TV	2	9.50	-	-
Radio	5	23.80	-	-
Church leader	3	14.30	-	-
**Preferred CC information delivery channels**			-	-
Verbal	21	100.00	-	-
Videos	2	9.50	-	-
Pictures	3	14.30	-	-
**Preferred settings for CC information dissemination**			-	-
Health facility	9	42.90	-	-
Anywhere	4	19.10	-	-
Community settings	2	9.50	-	-
Home	6	28.60	-	-

CHW, community health worker; CC, cervical cancer; HPV, human papillomavirus; TV, television; s.d., standard deviation.

**TABLE 2-A1:** Reasons for not screening for cervical cancer (*n* = 163 [unscreened women]).

Reason	*n*	%
Fear of the test outcome	9	5.2
Lack of information on CC screening	13	7.5
Not aware of the place to get screening service	18	10.3
The screening procedure is embarrassing	1	0.6
Absence of any symptoms/abnormality	57	32.8
Could not afford to pay for CC screening test	8	4.6
Long travel distance to the health facility with screening services	12	6.9
No reason or other	45	27.6

CC, cervical cancer.
